# Early-life stress reduces senescence in the field for an indeterminately growing ectotherm

**DOI:** 10.1093/evlett/qrag027

**Published:** 2026-07-02

**Authors:** Will Morgan, Hayley Cameron, Dustin Marshall

**Affiliations:** School of Biological Sciences, Monash University, Clayton, Victoria, 3800, Australia; School of Biological Sciences, Monash University, Clayton, Victoria, 3800, Australia; School of BioSciences, University of Melbourne, Parkville, Victoria, 3010, Australia; School of Biological Sciences, Monash University, Clayton, Victoria, 3800, Australia

**Keywords:** ageing, bugula neritina, invertebrate, life history, reproduction, senescence, survival

## Abstract

Theory predicts that aged-related declines in performance (senescence) depend on experiences during early life. Experimental field tests of this theory are rare: most manipulative studies have been done in the laboratory on short-lived invertebrates; while most field studies focus on longer-lived vertebrates and involve no experimental manipulation. Consequently, few studies manipulate early-life stress and test its effects on senescence in the field, especially in invertebrates. We manipulated early-life stress in the colonial marine bryozoan, *Bugula neritina*, by experimentally extending the larval period (an energetic stress), then monitored the survival, fecundity and growth of more than 500 individuals in the field across their lifetimes. We used these data to generate trajectories of actuarial and reproductive senescence and test how both components of ageing were affected by early-life stress. While colonial organisms with indeterminate growth such as *B. neritina* have been theorised to show negligible senescence, instead we saw strong declines in survival and reproductive output in the oldest individuals, regardless of early-life stress. We also found that adults originating from stressed larvae had lower fitness overall (in terms of lifetime reproductive success), but experienced weaker actuarial senescence. Our findings for reproductive senescence were more nuanced, with some evidence to suggest that early-life stress also decreases rates of reproductive senescence. Our study provides a rare demonstration of senescence for an invertebrate in the field and suggests that the relationship between life history and senescence may be more complex than has been anticipated.

## Introduction

Senescence is characterised by progressive declines in the probability of survival and fertility with age, termed actuarial and reproductive senescence, respectively (Gaillard & Lemaître, 2020). Historically, it was assumed that wild animals did not experience senescence because high extrinsic mortality risk prevented any individual from reaching ages where senescence could manifest (Kirkwood & Austad, 2000; Nussey et al., 2013). Evidence that actuarial and reproductive senescence occur in wild vertebrate populations is now widespread (Nussey et al., 2013), but not all species senesce (Jones et al., 2014). In some taxa, mortality and fertility rates are stationary (negligible senescence, e.g., Schaible et al., 2015), while theory and some data suggest that survival and fertility may even increase with age in some groups (negative senescence; Ebert, 2019; Vaupel et al., 2004). Documenting and explaining the diversity of senescence patterns in the wild remains an ongoing challenge in evolution and ecology.

Most field studies of senescence focus on endothermic vertebrates (mammals and birds) that are relatively large and have a tradition of long-term monitoring in the wild (e.g., Hayward et al., 2014; Millon et al., 2011; Nussey et al., 2007; Reed et al., 2008; Spagopoulou et al., 2020). Mammals and birds are determinate growers, a trait that is predicted to yield senescence (Vaupel et al., 2004), but this same theory base predicts that indeterminate growers (most ectotherms other than insects) should show no senescence. Most studies of senescence in ectotherms, particularly shorter-lived invertebrates, are laboratory-based, due largely to the difficulties of tracking small, mobile organisms in the wild (Zajitschek et al., 2020; but see: Bonduriansky & Brassil, 2002). Such laboratory studies can reduce extrinsic mortality, and organisms are often provided food *ad libitum*, both of which may distort rates of senescence (Mautz et al., 2019; Promislow et al., 2022). It therefore remains unclear whether the senescence observed in determinate growers in the wild also occurs in field populations of indeterminate growers.

Experiences early in life appear to shape senescence trajectories but again, our understanding of these effects is limited (Lemaître et al., 2015). For example, poor nutrition or stress early in life can lead to greater senescence at old ages for some species (Nussey et al., 2007; Reed et al., 2008) but may reduce senescence in others (Angell et al., 2020; Hooper et al., 2017). Field studies avoid the problems associated with laboratory environments described above by placing organisms in realistic conditions, but these studies have their own limitations (Bliard et al., 2025; Nussey et al., 2008). Field studies that seek to link senescence to early life history often rely on correlations, whereby senescence rates depend on whether individuals naturally experienced good or poor conditions early in life (e.g., lived in a population with abundant or limited resources; Bouwhuis et al., 2010; Fay et al., 2018; Tung et al., 2016; but see: Boonekamp et al., 2014). A potential problem with this correlative approach is that environmental conditions early in life often covary with conditions later in life (e.g., birds that experience a good juvenile habitat may secure good habitats as adults; van de Pol et al., 2006), or with the genetics of a given subpopulation. As such, manipulative tests of causative links between early life-history experiences and senescence, particularly in field populations of invertebrates, remain rare (but see: Angell et al., 2020).

Here we conduct a manipulative field test to determine how early-life experience affects actuarial and reproductive senescence in the colonial marine invertebrate, *Bugula neritina*—a species with indeterminate growth and a sessile adult stage that provides a tractable system for monitoring lifetime performance in the field. After manipulating the stress that larvae experienced (see Methods), we monitored the survival, reproduction and growth of each individual colony weekly across their lifetime (~6 months). From this data we generated complete trajectories of actuarial and reproductive senescence for each colony, while also investigating other important life history traits.

## Methods

### Study species

*Bugula neritina* is an arborescent bryozoan found in marine fouling communities worldwide. *B. neritina* is colonial and grows indeterminately through the asexual budding of modules (zooids) that regularly bifurcate from distal tips to form new branches (Cameron et al., 2016). Colonies produce larvae in specialised reproductive zooids (ovicells; Keough & Chernoff, 1987) that are released into the water-column. These larvae are non-feeding and depend on maternally provided energy reserves during swimming, settlement and metamorphosis. As such, delaying larval settlement results in energetic stress that can compromise subsequent adult performance (Wendt, 1996, 1998), but the impacts of delayed settlement on rates of senescence have gone unexplored.

### Broodstock collection, storage and spawning

To obtain larvae for our experiments, we collected adult *B. neritina* colonies from the Royal Brighton Yacht Club in Port Phillip Bay, Victoria, Australia in 2023. These colonies were stored in the laboratory in complete darkness in oxygenated, insulated aquaria. After 48 hours, we induced adult colonies to spawn their brooded larvae by exposing these colonies to bright light (Cameron et al., 2016). We spawned multiple colonies simultaneously and pooled the released larvae to ensure that we had sampled from a genetically diverse population.

### Experimental design

To determine the effect of larval stress on senescence in *B. neritina*, we experimentally extended the duration of the larval period. We used three levels of larval duration that represent a gradient of energetic stress: an undelayed larval period, where larvae were allowed to settle immediately after release from their parents (i.e., 0 hrs of enforced swimming); and two delayed larval periods, whereby larvae were forced to continue swimming for 6 or 18 hours before being presented with a settlement surface (Figure S1). We chose these larval periods to reflect those that naturally occur in the field within sublethal limits (Burgess & Marshall, 2011). We enforced larval swimming by placing the larvae in 50-mL tubes filled with filtered seawater that were rolled continuously (40 RPM) under fluorescent lights.

### Larval settlement and field deployment

After preventing larval settlement for the appropriate duration, we collected individual larvae in glass pipettes and placed these larvae onto roughened acetate sheets within a drop of seawater where they were allowed to settle in the dark. After 1 hour, any unsettled larvae were removed by carefully rinsing the acetate sheets with seawater (i.e., all larval treatments had up to 1 additional hour to settle). The acetate sheets (bearing successful settlers) were then submerged in trays of seawater overnight. During this time settlers begin metamorphosis into the adult form.

The next day we cut around the settlers on the acetate sheets and attached them to PVC plates (5cm x 5cm) at a density of four settlers per plate with cyanoacrylate superglue. We then stored these plates (and the attached larvae) in insulated, oxygenated aquaria overnight prior to field deployment at the Royal Brighton Yacht Club. Settlement plates were randomly attached to three PVC backing panels (55 × 55 × 0.8cm) and hung facedown ~1.5m below the surface.

The entire experiment was done once in June, and once in July 2023 (two experimental blocks). In each block, three PVC backing panels were deployed, each bearing ~22 plates (Figure S1) so that, overall, we deployed 536 experimental colonies across 134 plates (n = 152 colonies for 0-hours, 152 colonies for 6-hours and 232 colonies for 18-hours). Plates and panels were cleaned weekly to remove non-experimental organisms.

### Data collection

We measured the performance of colonies in the field every week until all colonies had died (June: 25 weeks; July: 22 weeks). We used well-established approaches for this species to monitor survival, growth and reproduction over the experimental period (see Cameron et al., 2016; Keough & Chernoff, 1987 for details). Survival was determined by the presence of the focal colony attached to the plate (scored as 1), while we assumed that absent individuals had died (scored as 0). Colony growth was measured as the number of bifurcations along the longest branch—a reliable measure of colony size in this species (Keough & Chernoff, 1987). We assessed reproductive onset each week, and once colonies became reproductive, we counted the number of active ovicells on each colony with a dissecting microscope at 10X magnification. When the fecundity of a colony exceeded 500 ovicells we subsampled one major branch of the colony and multiplied this count by the number of major branches.

### Analytical methods

Analyses were performed using RStudio (v4.4.1; R Core Team, 2024) and the packages lme4 (v1.1.35.3; Bates et al., 2015), lmerTest (v3.1.3; Kuznetsova et al., 2017), glmmTMB (v1.1.14; Brooks et al., 2017), flexsurv (v2.3.2; Jackson, 2016), car (v3.1.3; Fox & Weisberg, 2019) and emmeans (v1.10.4; Lenth, 2024). Plots were created using ggplot2 (Wickman, 2016) and patchwork (Pedersen, 2024). Assumptions were tested using DHARMa (v0.4.6; Hartig, 2022) and custom R scripts. In all cases we first fit full models that included all interactions between our fixed effects and subsequently reduced these models by removing interactions where appropriate (Quinn & Keough, 2002). Microsoft Copilot was used to diagnose errors in RStudio code.

### Actuarial senescence

We analysed differences in actuarial senescence—the increase in mortality rate with age—using two methods. First, we used parametric survival models—an approach with a long tradition in studies of senescence. Survival models have the benefit of generating familiar parameter estimates that describe the nature and magnitude of senescence and thus are directly comparable across studies of senescence (see below for details). But parametric survival approaches are insufficiently complex to explicitly account for spatial and temporal (co)variances in survival—features that are common to field studies (including in our system: Cameron et al., 2016; Schuster et al., 2021). As such, we also analysed the probability of survival over time using repeated-measures logistic ANCOVAs that can explicitly account for these covariances via the inclusion of nested random effects. Thus, although the parameter estimates derived from a logistic approach are less grounded in the theory of senescence, they provide more reliable tests for differences in slopes and intercepts among larval period treatments over and above spatial and temporal (co)variances. Because both analytical approaches have advantages and disadvantages, we used both to ensure that our findings were robust, comparable, and applicable to data from field studies. We describe and present the results for both methods below.

For our parametric survival approach, we first fit five survival distributions (exponential, Gompertz, 2-parameter Weibull, log-normal and log-logistic) to data on colony lifespan (the number of weeks each colony lived). Larval period and deployment month had either additive or interactive effects, and model selection was performed using AIC and likelihood ratio tests where appropriate (Quinn & Keough, 2002). If competing models had similar AIC values (ΔAIC < 2), then the most parsimonious was selected. An additive model (i.e., no larval period x deployment month interaction) with a Gompertz distribution consistently provided the best fit to our data (Table S1). In the Gompertz model, the rate parameter (a) describes the initial or juvenile mortality rate, while the shape parameter (b) describes the exponential age-specific increase in mortality rate (Sherratt et al., 2010). The additive Gompertz model was then compared to models that systematically removed the effects of larval period or deployment month on both the rate (a) and(or) shape (b) parameters. Stepwise model selection converged on an additive model that included both larval period and deployment month, and that fit unique shape parameters (b) for each larval period (see Results and Table S2 and S3). Note that in the Gompertz model, age-specific mortality rates can decrease (b < 1, i.e., negative senescence), remain constant (b = 1, i.e., constant or negligible senescence) or increase (b > 1, i.e., senescence) over time—providing a direct test of the nature and strength of senescence. After estimating the shape parameter (b) for each larval period, we used Wald tests to assess whether these estimates differed significantly from the null case of negligible senescence (i.e., H_0_: b = 1; H_a_: b ≠ 1).

For our logistic ANCOVA approach, we used generalised linear-mixed models with binomial error distributions and logit-link functions where survival was a binary response variable (1 = alive; 0 = dead); larval period and month of deployment were categorical fixed effects; and colony age was a continuous fixed effect. We specified random intercepts for each colony (to account for repeated measures), and for plate nested within panel (to account for the non-independence of colonies on each plate). Note in our logistic approach, the main effect of larval period tests for differences in early (juvenile) mortality; while a larval period x age interaction tests for differences in age-specific mortality rates (i.e., slopes)—thus these tests are analogous to those for the rate (a) and shape (b) parameters in the parametric survival analyses, respectively. Importantly, our logistic approach revealed significant effects of larval period on both age-specific (slopes) and juvenile (intercepts) mortality—consistent with our findings from the parametric survival analysis (see Results). Congruence between the two approaches thus strengthened our confidence in our findings, despite the limitations of both approaches (discussed above).

Although our logistic ANCOVA approach revealed that larval period affected age-specific mortality rates (i.e., larval period x age interaction) across the entire experimental period, the slopes obtained from this analysis provide insufficient information to make robust inferences about how the larval period altered mortality rates in old age (i.e., senescence). As such, we also fit separate logistic models that examined survival in the adult period only (i.e., after each colony became reproductive) to isolate these effects. These models had the same structure as previously described (for the logistic ANCOVA across the entire lifespan).

### Reproductive senescence

To investigate reproductive senescence, which we define as a decrease in reproductive output with age, we again conducted two analyses: one that examined the trajectory of reproduction over the entire lifespan; and another that investigated the rate at which reproduction declined in old age relative the maximum reproductive output of the colony. We used a combination of these two approaches as the first allows us to estimate if absolute senescence rates vary between larval period treatments; the second allows us to estimate relative senescence rates when other covarying life-history traits (i.e., differences in size; see Supplementary materials for details) are accounted for—an approach that is useful from a comparative perspective (Baudisch & Stott, 2019).

Reproductive trajectories were quadratic in all cases (as determined using maximum likelihood estimation; see Results): reproduction increased from zero, then plateaued before declining near the death of the colonies. Reproduction was analysed using linear-mixed effects models where log-transformed weekly ovicell counts was the response variable (note that log-transformed data better adhered to model assumptions over GLMs that included other error distributions). In these analyses, larval period and deployment month were categorical fixed effects; colony age was modelled with a quadratic term and as a continuous fixed effect; and colony ID and plates nested within panel were again included as random intercepts.

We explored rates of reproductive senescence by testing for differences in slopes that described the linear decline in reproduction as colonies aged. Note that growth differed between larval periods—with undelayed larvae reaching larger sizes and accumulating more ovicells per bifurcation (Figure S2, S3 and Table S4). As such, larger colonies had greater absolute age-specific declines in reproduction compared to smaller colonies. To account for this size effect, we standardised weekly reproductive decline for each colony by dividing weekly measures of fecundity by the number of ovicells present at the time of maximum reproduction for that colony. Thus, relative reproduction varied between 1 and 0, where 1 indicates maximum reproductive output and 0 represents no reproduction. We then analysed this metric of standardised reproduction as a function of time since peak reproduction. Larval period and month of deployment were categorical fixed effects, time was a continuous fixed effect, and both colony ID and plate nested within panel were included as random intercepts. Only colonies that become reproductive were included in these analyses (i.e., we excluded all colonies that died before reproductive maturity).

Finally, we explored differences in lifetime reproductive output across our larval period treatments since reproductive effort is sometimes correlated with rates of senescence (Lemaître et al., 2015). To estimate lifetime reproductive output, we summed the number of ovicells present on the colonies at each time point across their lifespan to obtain a cumulative measure of fecundity for each colony (note that in *B. neritina*, ovicells are reused over time). We then analysed cumulative fecundity using a generalised linear mixed-model with a negative binomial distribution (accounting for zero-inflation) and log-link function, where larval period and deployment month were categorical fixed effects and plate nested within panel was a random effect. We again only included colonies that survived until reproductive maturity.

## Results

### General performance

Larval experience affected all aspects of adult performance, including survival, reproduction and growth. Individuals deployed in June outperformed those deployed in July (Table S5), but the effects of larval period were generally consistent across deployment months (Table S2 and S6). We thus present analyses pooling across months of deployment below (unless otherwise stated).

### Actuarial senescence

*Bugula neritina* consistently experienced strong actuarial senescence in the field (Figure 1)—mortality rates increased with age regardless of larval period (i.e., Gompertz shape parameters (b) for all larval periods > 1 [overall b = 1.124 (95% CI: 1.106–1.142]; Table 1; Figure 1A). Larval stress affected age-specific mortality rates in both analytical approaches we used (LRT comparing Gompertz models with and without larval period specific shape parameters (b): χ^2^ = 26.814, df = 2, *P* < 0.001; logistic regression of survival across the entire lifespan: larval period x colony age: χ^2^ = 20.058, df = 2, *P* < 0.001). As adults, colonies that experienced shorter larval periods (i.e., less-stressful early lives) died at higher rates than those from longer larval periods (logistic regression for post-maturation survival: larval period x colony age: χ^2^ = 51.163, df = 2, *P* < 0.001; Table 1). In other words, colonies that experienced less stress early in life stress showed more actuarial senescence in old age (Figure 1A; Table 1).

**Figure 1:**
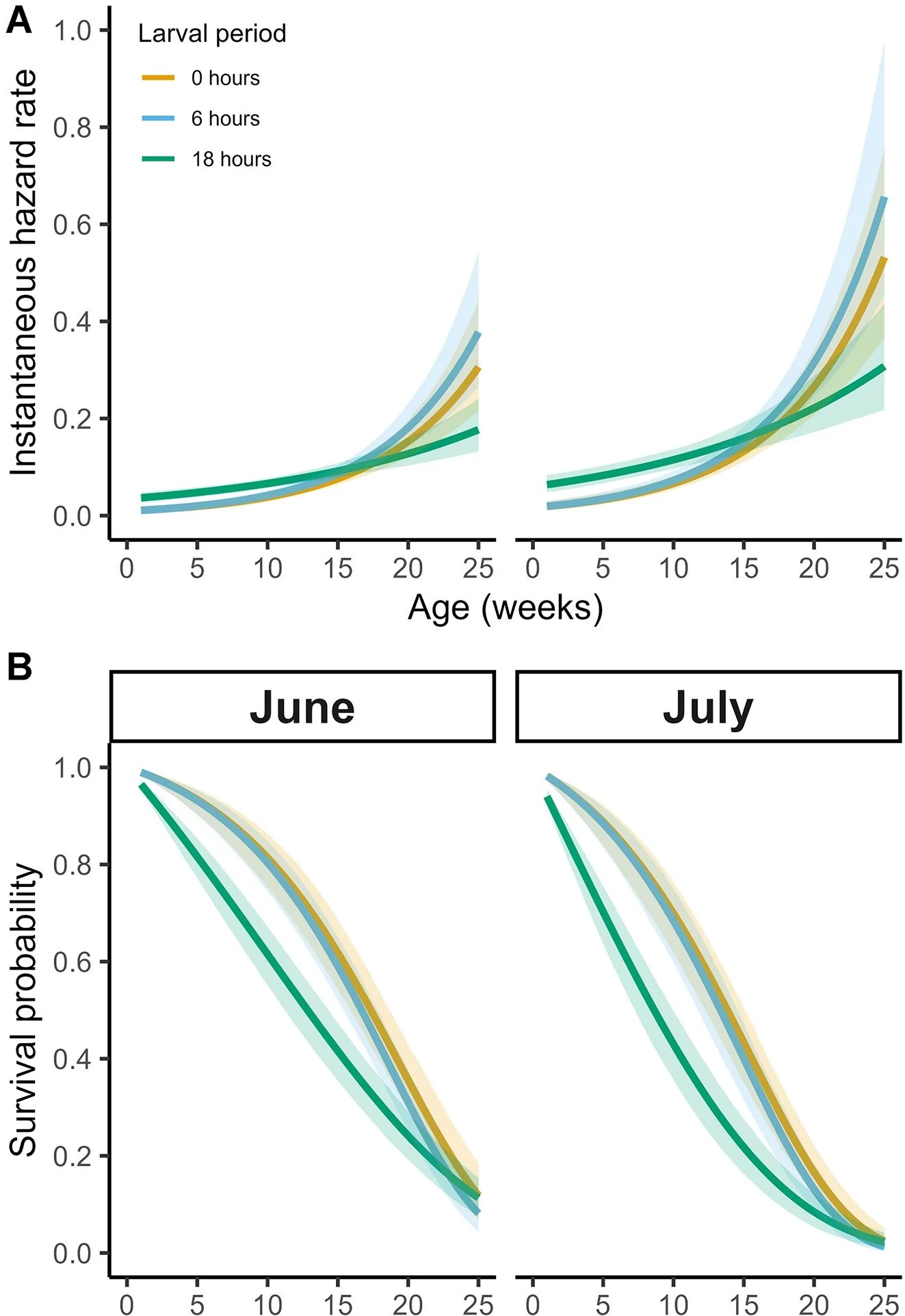
The effects of early-life stress (larval period) on the (A) the instantaneous hazard rate and (B) probability of survival of *Bugula neritina* colonies across their lifetime in the field. In both panels the coloured lines show the predicted values over time; the shaded areas show the 95% confidence intervals.

**Table 1:** Parameter estimates from the best fitting Gompertz model (additive with unique shape parameters for each larval period). Estimates are on the log scale and show deviation from the reference group^a^. Exponentiated estimates (exp(Estimate)) show the value of parameter estimates^b^ on the natural scale (shown in bold). Larger values for rate (a) indicate higher initial or juvenile mortality. Exponentiated estimates for age-specific mortality rate indicate negative senescence (b < 1), negligible senescence (b = 1) or senescence (b > 1). Note that when b > 1, larger values indicate higher rates of actuarial senescence. Results for Wald tests (H_0_: b = 1; H_a_: b ≠ 1) on larval period shape parameters are included.

Parameter	Variable	Estimate (95% CI)	SE	exp(Estimate)	z	*P*
Rate (a)	*Larval period*					
	0 hours^a^	0.017 (0.010, 0.026)	0.004	**1.016**		
	6 hours	0.010 (-0.631, 0.650)	0.330	**1.026**		
	18 hours	1.290 (0.756, 1.820)	0.270	**3.677**		
	*Deployment month*					
	June	-0.552 (-0.728, -0.377)	0.090	**0.585**		
Shape (b)	*Larval period*					
	0 hours^a^	0.139 (0.109, 0.169)	0.015	**1.149**	9.178	<0.001*
	6 hours	0.008 (-0.034, 0.050)	0.021	**1.158**	9.556	<0.001*
	18 hours	-0.073 (-0.109, -0.037)	0.018	**1.067**	6.160	<0.001*

a All estimates are compared to reference colonies with larval period = “0 hours” and month of deployment = “July”.

b Gompertz hazard function: ${{\mu }_x} = a{{e}^{bx}}\ $(Sherratt et al., 2010).

* Indicates *P* < 0.05

In contrast, colonies that experienced more stressful early lives (i.e., longer larval periods) experienced greater juvenile mortality (LRT comparing Gompertz models with and without the effect of larval period on juvenile mortality rate (a): χ^2^ = 9.955, df = 2, *P* = 0.007; main effect of larval period from logistic regression: χ^2^ = 48.826, df = 2, *P* < 0.001; Table 1). About 50% of colonies from longer larval periods died before adulthood, while only ~30% of those from shorter larval periods died during the same time (Figure 1B).

### Reproductive senescence

Regardless of larval experience, reproduction followed a quadratic trajectory with respect to colony age (maximum likelihood: χ^2^ = 1749.1, df = 4, *P* < 0.001)—colonies began reproduction at ~ 10 weeks old; reproduction peaked and then declined (Figure 2A; see also Figure S4). Thus, all colonies underwent reproductive senescence. However, reproductive senescence was stronger in colonies that had shorter larval periods (larval period x age^2^: F_2,2380.47_ = 7.754, *P* < 0.001; Table S7).

**Figure 2:**
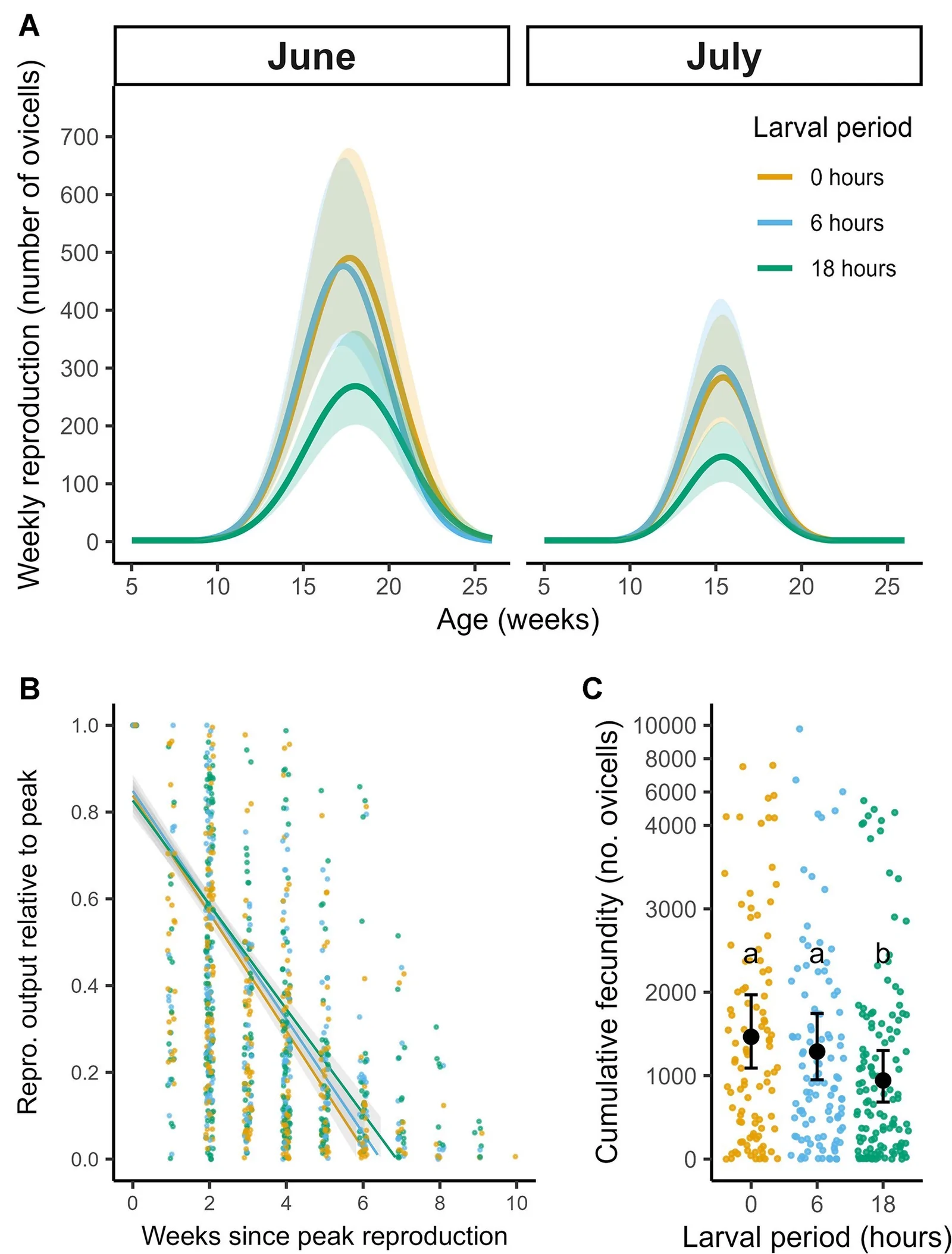
The effects of early-life stress (larval period) on the (A) reproductive trajectories; (B) standardised rate of reproductive decline and (C) lifetime reproductive output (cumulative fecundity) of *Bugula neritina* colonies in the field. In A and B, solid lines show predicted values from the best-fitting models and shaded areas are the 95% confidence intervals (CIs; estimated via parametric bootstrapping using 500 simulations for A). In C the large dots show values of mean (± 95% CIs) cumulative fecundity and lettering indicates significant differences (see Table S8). In all plots, small dots show the raw datapoints for each individual colony.

While the above analyses indicated that all larval periods underwent reproductive senescence, adults that had shorter larval periods appeared to have greater senescence (i.e., stronger reproductive declines with age). However, this effect only emerged because adults that experienced shorter larval periods had greater maximum reproductive outputs (i.e., compare reproductive peaks across larval periods in Figure 2A), such that their subsequent rates of reproductive decline appeared steeper. Accordingly, when we standardised the reproductive output of each colony by their maximum reproductive output, rates of reproductive senescence did not differ according to larval period (larval period x time since peak reproduction: F_2,1121.79_ = 1.570, *P* = 0.209; Figure 2B).

Finally, colonies that experienced shorter larval periods produced about 45% more ovicells (~450 ovicells) over their lifetime than colonies from longer larval periods (χ^2^ = 13.111, df = 2, *P* = 0.001; Figure 2A, C; Table S8)—in other words, a less-stressful early life led to higher lifetime reproductive output. Taken together then, our findings suggest that colonies that experience less stressful early lives have higher fitness overall (in terms of lifetime reproductive success) but experience greater rates of actuarial and absolute reproductive senescence.

## Discussion

We found that *Bugula neritina* experienced both actuarial and reproductive senescence—thus, our work provides a rare example of an invertebrate with indeterminate growth undergoing senescence in the field. We also found that while adults that experienced greater early-life stress (i.e., a longer larval period) had lower fitness overall (in terms of early survival, lifetime reproductive output and growth), these poorly performing adults had lower rates of actuarial senescence later in life. Meanwhile, early-life experience had more nuanced effects on the rate of reproductive declines with age. Absolute reproductive declines were greatest in colonies from shorter larval periods (i.e., indicating more reproductive senescence) but when reproductive declines were standardised relative to maximum reproductive output, we found that rates of reproductive senescence were consistent between larval periods. Our results suggest that the relationship between senescence and life-history varies at both intra- and interspecies scales. Accurately predicting senescence trajectories in underrepresented taxonomic groups will likely require further experimental study.

*Given that B. neritina* is a colonial ectotherm with indeterminate growth, it was surprising to observe a strong signal of senescence overall. Theory predicts that senescence should be relatively weak in such organisms: Vaupel et al. (2004) argue that indeterminate growers whose reproductive output scales with body size (as observed here; see Figure S2), should show negligible or even negative senescence. Negative senescence has been recorded in other marine invertebrates with these traits (e.g., sea urchins: Ebert, 2008, 2019; corals: Babcock, 1991; Montero-Serra et al., 2018). We suspect that the “weedy” life history of *B. neritina* (an invasive species with a rapid pace of life: Lagos et al., 2017; McMahon, 2002) may explain our observation of senescence in this species relative to other colonial organisms with slower life histories (e.g., corals). Indeed, faster life histories are associated with greater rates of senescence in endotherms (Jones et al., 2008) and a similar covariance may exist in invertebrates—though this requires further tests (Montero-Serra et al., 2018). Our findings also contradict classic theory specific to colonial organisms that emphasises size over age as the dominant driver of demography (Cameron et al., 2019; Caswell, 1988; Dahlgren & Roach, 2017; Hughes, 1984; Kirkpatrick, 1984). We join others in suggesting that both size and age must be considered when modelling the demography of colonial organisms (Hart & Keough, 2009) otherwise the contribution of older, larger adults could be overestimated. For now, and contrary to theoretical predictions, it appears that neither coloniality, indeterminate growth, nor ectothermy are perfect safeguards against senescence.

We found that adults that experienced less stress early in life (i.e., shorter larval periods) showed higher rates of actuarial senescence later in life. Stronger actuarial senescence may be common in invertebrates that experience less stressful larval conditions. In both neriid (*Telostylinus angusticollis*) and antler flies (*Protopiophila litigata*), high-quality larval diets (analogous to the reduced energetic stress of a shorter larval period) were associated with increased actuarial senescence (Adler et al., 2016; Angell et al., 2020; Hooper et al., 2017). Dietary restriction reduces ageing in many species (Nakagawa et al., 2012), and such effects seem at least possible in *B. neritina*. Longer larval periods in *B. neritina* can affect nutrition in two ways: 1) by reducing larval energy reserves that may be carried over to the adult phase after metamorphosis (Wendt, 2000); and 2) resulting in adults that have smaller feeding structures, and presumably lower rates of food intake (Best & Thorpe, 1986; Burgess & Marshall, 2011; Wendt, 1996). Both of these nutritional effects of a longer larval period could (perhaps counter-intuitively) result in decreased rates of senescence as we observe here. An interesting next step would be to determine whether alleviating nutritional stress (i.e., via supplementary feeding of adults as per Svensson & Marshall, 2015) increases senescence rates following an extended larval period—if so, these findings would support our hypothesis that larval duration mediates senescence via energetic pathways.

The Disposable Soma Theory of Ageing (Kirkwood & Austad, 2000; Kirkwood & Holliday, 1979) offers a second possible explanation for why colonies that experienced less stress as juveniles showed higher rates of senescence. This theory suggests that the physiological costs of increased investment in reproduction should accelerate ageing (Kirkwood, 2005; Kirkwood & Rose, 1991). In our study, adults that experienced shorter larval periods had lifetime reproductive outputs that were ~45% higher than those that experienced longer larval periods—thus these colonies may experience a life-history trade-off, whereby greater investment into reproduction comes at the expense of somatic maintenance, and hence increased senescence rates.

It is also worth noting that we cannot discount the role of selective disappearance in driving our results (van de Pol & Verhulst, 2006). Indeed, in manipulating the larval period, we may have inadvertently selected for well-provisioned larvae that swim for longer and often perform better as adults (Marshall et al., 2003) and thus may be pre-disposed to lower senescence in adulthood. Indeed, larval mortality in the laboratory was higher for those forced to swim for 18-hours (c.f. 6-hours; W. Morgan, personal observation). These effects may have also compounded over the experiment as longer larval periods increased juvenile mortality, potentially further purging lower-quality individuals that may inherently have stronger senescence.

In contrast to patterns of actuarial senescence, the effects of larval stress on reproductive senescence were nuanced. Reproductive trajectories indicated that shorter larval periods increased absolute rates of senescence but, when considered relative to peak reproductive output, senescence rates were unaffected by larval period. Absolute measures of reproductive decline reflect the true fate of each individual colony and so are perhaps more informative for making inferences about the impacts of senescence on organismal fitness and(or) demography (Cameron et al., 2019; Coulson et al., 2010). However, relative measures of senescence have been argued to yield more rigorous comparisons across groups as they account for life history variation (Baudisch & Stott, 2019) and so may reveal underlying factors that influence senescence independently of the environment. For example, that larval period did not influence relative rates of reproductive senescence suggests that age-specific reproduction may be somewhat conserved (i.e., have a genetic component; Wilson et al., 2008). Whether our results are representative of patterns observed across broader taxa is unclear given the paucity of data from indeterminately growing ectotherms—demonstrating the need for future studies in diverse, non-model species. Overall then, our study highlights the need for more manipulative tests of senescence in the field as our findings were largely unanticipated by current theory.

## Supplementary Material

qrag027_Supplemental_File

## Data Availability

The data and code used in this study are available from the Figshare Digital Repository: https://doi.org/10.26180/31175779 (Morgan et al., 2026).
