# Supplementary material for: Early-life stress reduces senescence in the field for an indeterminately growing ectotherm
Source: Evol Lett. 2026 Jul 2;10(5):483–92. doi: 10.1093/evlett/qrag027 (PMC13629228; doi:10.1093/evlett/qrag027)
Supplement: Supplementary file 1 [file qrag027_supplemental_file.pdf]

Supplementary Material for:

Early-life stress reduces senescence in the field for an indeterminately growing ectotherm

Evolution Letters

Will Morgan, Hayley Cameron and Dustin Marshall

Corresponding author: Will Morgan, will.morgan1@monash.edu

Contact Author: Will Morgan, will.morgan1@monash.edu

The file includes:

Full description of analytical methods for growth

Full description of the results for growth

Tables S1-S8

Figure S1-S4

Literature cited in the appendix

Supplementary analytical methods for growth

We investigated colony growth as, like reproduction, faster growth is often correlated with increased senescence (Lemaître et al. 2024). The weekly growth of each colony was analysed using a linear mixed effects model. Colony size (in bifurcations) was a continuous response; colony age was a continuous predictor; larval period and month of deployment were included as categorical predictors. We included a random effect of colony ID to account for repeated measures and a random effect of plate nested within panel. Growth was only analysed up to week 23; after this time, there was insufficient data within each larval period for our models to remain reliable. Deployment month had significant effects on weekly growth when fitting full models (Table S4 and S5). We therefore fit identical models (but without the effect of deployment month) to the same data within each month.

To determine the effect of larval period on maximum colony size, we used linear mixed effects models that included maximum size as a continuous response variable, and larval period and month of deployment as categorical predictors. A random intercept of plate nested within panel was included.

The relationship between colony size and reproductive output was analysed using a linear-mixed effects model. Ovicell number was a continuous response, while colony size was a continuous predictor. Larval period and deployment month were included as categorical predictors. Since we recorded ovicell counts and colony size weekly, each colony had multiple data points relating ovicell counts to size. We therefore included random intercepts for colony ID to account for these repeated measures and a random effect of plate nested within panel.

Supplementary results for growth

B. neritina colonies grew more and achieved the largest maximum sizes when they had experienced a short larval period (Figure S4). The effect of larval period on growth patterns was dependent on deployment month (larval period x age x deployment month: $F_{2, 6080.2} = 9.930$, $p < 0.001$; Figure S4). Adults that experienced shorter larval periods grew faster in June (larval period x age: $F_{2, 3604.4} = 13.100$, $p < 0.001$), but not in July (larval period x age: $F_{2, 2440.6} = 2.016$, $p = 0.133$). When adults were nearing the end of life, colony sizes converged for

all larval periods (Figure S4). Size convergence was driven by size-specific mortality in the largest adults, giving the impression that adults shrank in the final weeks. Maximum colony size was similarly dependent on deployment month and larval experience (larval period x deployment month: $F_{2, 378.5}= 4.423$, $p= 0.013$). In June, all colonies reached similar maximum sizes (~12 bifurcations; Table S8), while in July colonies from shorter larval periods were about 3 bifurcations larger (~4500 zooids) than colonies from longer larval periods (Figure S4; Table S8).

Larger colonies always produced more ovicells ($F_{1, 2631.33}= 396.00$, $p< 0.001$; Figure S2). However, as colonies grew those that that had experienced less stress as larvae (i.e. shorter larval periods) produced more ovicells than those that had experienced greater stress early in life (larval period x colony size: $F_{2, 2633.85}= 7.934$, $p< 0.001$; Figure S2). 0- and 6- hour colonies increased reproduction by ~42 ovicells per bifurcation; 18-hour colonies only increased reproduction by ~29 ovicells for the same increase in size.

Table S1. Results of survival distribution selection using AIC. We fit two types of models: additive main effects models (i.e. larval period + deployment month) and interactive main effects models (i.e. larval period + deployment month + larval period:deployment month). A model with AIC ≥ 2 lower than any other model was considered a better fit to our data. In the case of model ties, we selected the most parsimonious model and confirmed our choice using LRT.

	Additive models AIC	Interaction models AIC
Gompertz	3616.509*	3616.811
2-parameter Weibull	3808.339	3809.961
Exponential	3824.082	3826.152
Log-normal	3953.083	3955.734
Log-logistic	3989.660	3989.552

*Indicates best fitting model

Table S2. AIC values for additive parametric survival models fit using a Gompertz distribution. A model with AIC ≥ 2 lower than any other model was considered a better fit to our data.

Model formula	AIC
Larval period + deployment month	3616.509
Larval period	3652.228
Deployment month	3622.464
Larval period + deployment month with larval period specific shape parameters	3593.695*
Larval period + deployment month with deployment month specific shape parameters	3599.802
Larval period:deployment month with larval period specific shape parameter	3596.627

*Indicates best fitting model

Table S3. Results of likelihood ratio tests that compare models of the effects of larval period and deployment month on actuarial senescence in *Bugula neritina*. Each stepwise test includes the rationale for the test. Models are described according to the main effects included. Significance indicates that the second model listed provides a better fit to the data.

Model comparison	χ^2	DF	p	Rationale
Larval period + deployment month vs. Larval period:deployment month	3.697	2	0.157	Does an interaction or additive model provide a better fit to the data?
Larval period + deployment month vs. Larval period + deployment month with larval period specific shape parameter	26.814	2	<0.001*	Are rates of actuarial senescence different between larval periods?
Larval period + deployment month with larval period specific shape parameter vs. Larval period:deployment month with larval period specific shape parameter	1.068	2	0.586	Does the additive model still provide the best fit to the data when including larval period specific shape parameters?
Deployment month vs. Larval period + deployment month	9.955	2	0.007*	Is the rate parameter (a) of the Gompertz model influenced by larval period?
Larval period vs. Larval period + deployment month	64.533	1	<0.001*	Is the rate parameter (a) of the Gompertz model influenced by deployment month?
Larval period + deployment month vs. Larval period + deployment month with deployment month specific shape parameter	18.707	1	<0.001*	Are rates of actuarial senescence different between deployment months?

*Indicates $p < 0.05$

Table S4. Significance of maximum size differences between larval periods split by deployment month measured in bifurcations.

Contrast	Estimate	SE	df	t	p
June					
0 – 18 hours	0.438	0.847	409	0.517	0.863
0 – 6 hours	0.621	0.886	401	0.700	0.763
18 – 6 hours	0.183	0.754	337	0.243	0.968
July					
0 – 18 hours	3.680	0.842	408	4.370	<0.001*
0 – 6 hours	1.182	0.905	351	1.306	0.393
18 – 6 hours	-2.498	0.911	356	-2.742	0.018*

*Indicates p< 0.05

Table S5. Summary of the effect of month of deployment (block) on performance in *Bugula neritina* colonies averaged across larval period treatments. The direction of the effect is noted. See Methods for details on model fitting and selection.

Performance Metric	F or χ^2	DF	p	Effect
Adult actuarial senescence				
Deployment month x week	11.666	1	<0.001*	Weekly probability of death is ~60% higher in July compared to June
Reproductive trajectory				
Deployment month x week^2 (i.e. colony age^2)	157.161	2, 2376.32	<0.001*	Curvature is less steep in June
Reproductive senescence rate				
Deployment month x standardised week	1.577	1, 1099.96	0.210	
Cumulative fecundity				
Deployment month	6.670	1	0.010*	June colonies produce ~500 more ovicells over their lifetime
Maximum size				
Deployment month	20.699	1, 4.29	0.008*	June colonies ~2.5 bifurcations larger
Weekly growth				
Deployment month x week	8.903	1, 6082.3	0.003*	Weekly growth ~3% faster in June
Size dependent reproduction				
Deployment month x size	8.282	1, 2644.08	0.004*	June produces 11 more ovicells per increase in bifurcation

*Indicates p< 0.05

Table S6. Summary of the interaction between month of deployment (block) and larval period on performance of *Bugula neritina* colonies. See Methods for details on model fitting and selection.

Performance Metric	F or χ^2	DF	p
Adult actuarial senescence			
Deployment month x larval period x week	2.069	2	0.355
Reproductive trajectory			
Deployment month x larval period x week^2 (i.e. colony age^2)	2.703	2, 2416.5	0.067
Reproductive senescence rate			
Deployment month x larval period x standardised week	0.2807	2, 1114.69	0.755
Cumulative Fecundity			
Deployment month x larval period	1.003	2	0.606
Maximum size			
Deployment month x larval period	4.423	2, 378.50	0.013*
Weekly growth			
Deployment month x larval period x week	9.930	2, 6080.2	<0.001*
Size dependent reproduction			
Deployment month x larval period x size	0.645	2, 2637.40	0.525

*Indicates p< 0.05

Table S7. Fixed effects estimates and 95% confidence interval for log transformed weekly reproductive output in *Bugula neritina*. Coefficient estimates are derived from the fixed effects estimates of quadratic models[a] that relate weekly reproductive output to age for each larval period treatment. Coefficients are on the log scale and all estimates are compared to reference colonies with larval period = “0 hours”.

	a	b	c
Larval period			
0-hours	-0.119 (-0.126, -0.111)	3.661 (3.438, 3.884)	-23.267 (-24.820, -21.714)
6-hours	-0.006 (-0.014, 0.002)	0.158 (-0.070, 0.387)	-0.953 (-2.607, 0.701)
18-hours	0.008 (0.001, 0.015)	-0.248 (-0.463, -0.033)	1.233 (-0.334, 2.800)

[a] $y = ax^2 + bx + c$

Table S8. Significance of lifetime fecundity differences between larval periods in *Bugula neritina* colonies fit using a negative binomial model. Estimates are on the natural log scale.

Contrast	Estimate	SE	df	z	p
0 – 18 hours	0.441	0.126	-	2.861	0.001*
0 – 6 hours	0.129	0.122	-	1.052	0.544
18 – 6 hours	-0.313	0.121	-	-2.589	0.026*

*Indicates $p < 0.05$

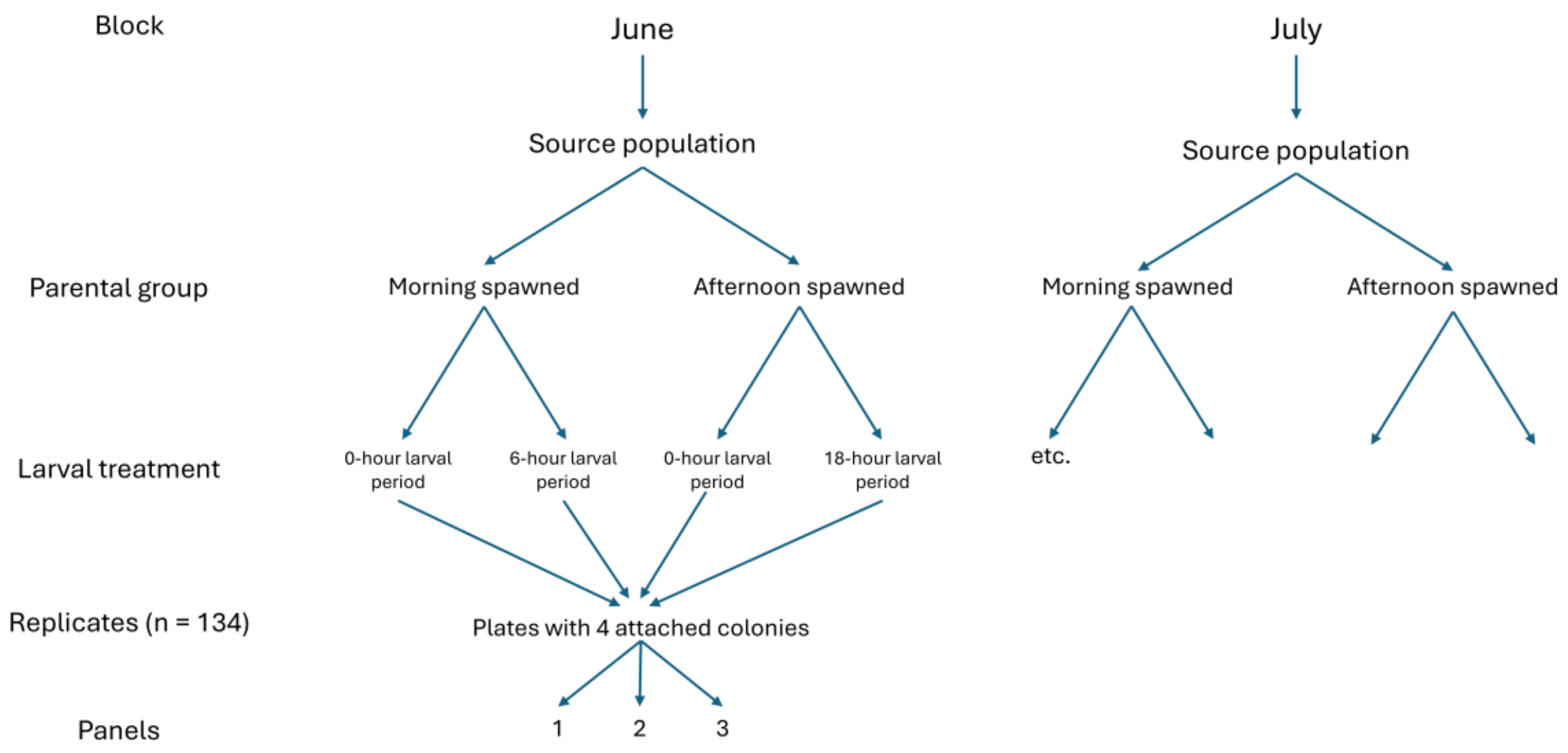

Figure S1. Schematic of the experimental design. The figure shows the outline of how larvae were gathered, manipulated and deployed to the field for the June block. The process was repeated in the July block. The number of replicate plates deployed in the June and July blocks were 69 and 65, respectively. All plates in each block were distributed across 3 panels (total panels = 6). To account for potentially confounding differences in the timing of larval spawning between larval period treatments (Kosman and Pernet 2009), we spawned different batches of adult colonies in the morning and the afternoon. Larvae spawned from colonies in the morning were assigned to 0 and 6-hours larval periods; those spawned in the afternoon were assigned to either a second 0-hour group or the 18-hour larval period. We were thus able to orthogonally control the timing of larval spawning to explore the effects of larval period on subsequent performance.

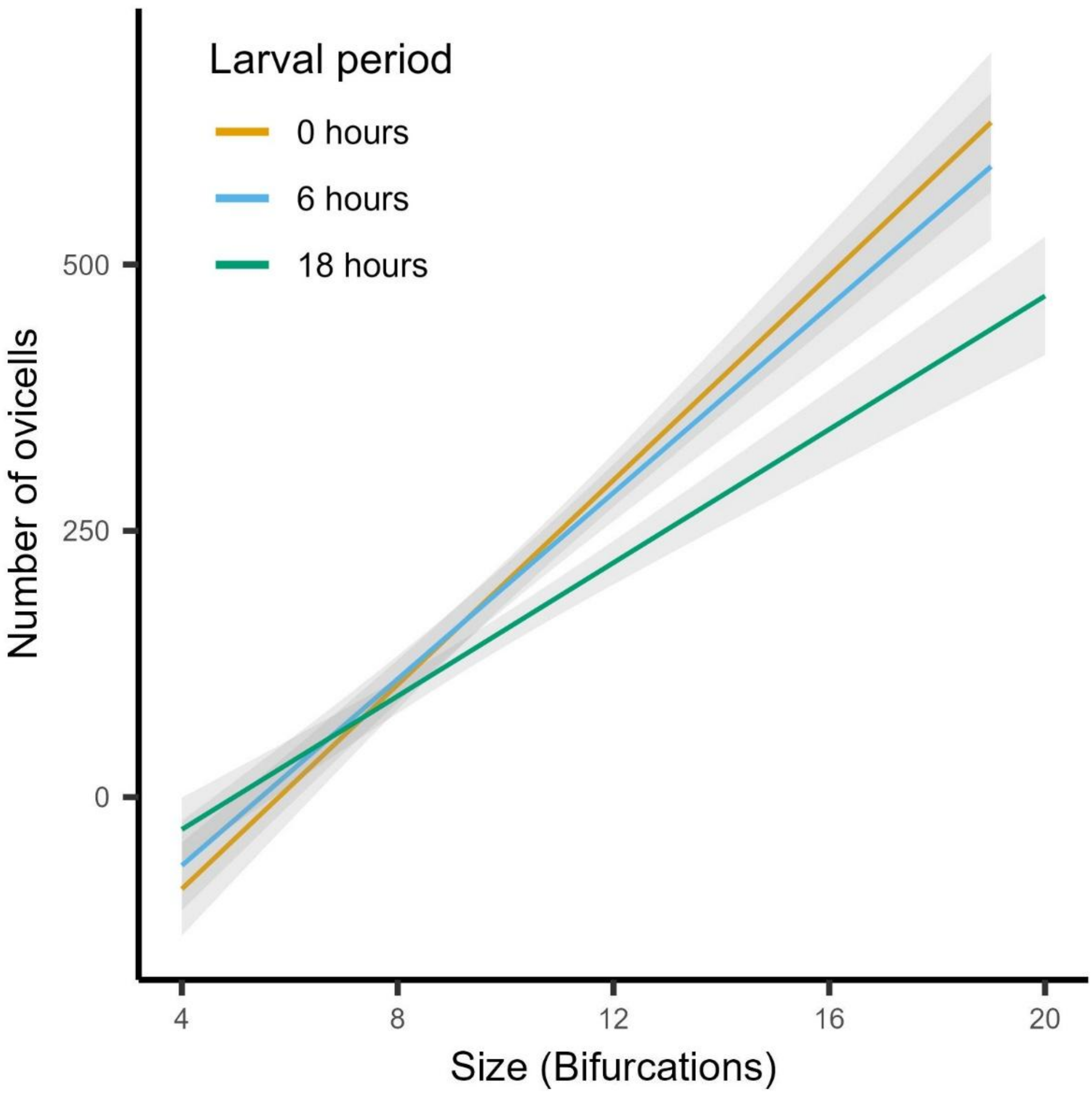

Figure S2. The effect of size on the number of ovicells in *Bugula neritina* colonies. Coloured lines show the line of best fit, and shaded areas shows the 95% confidence interval.

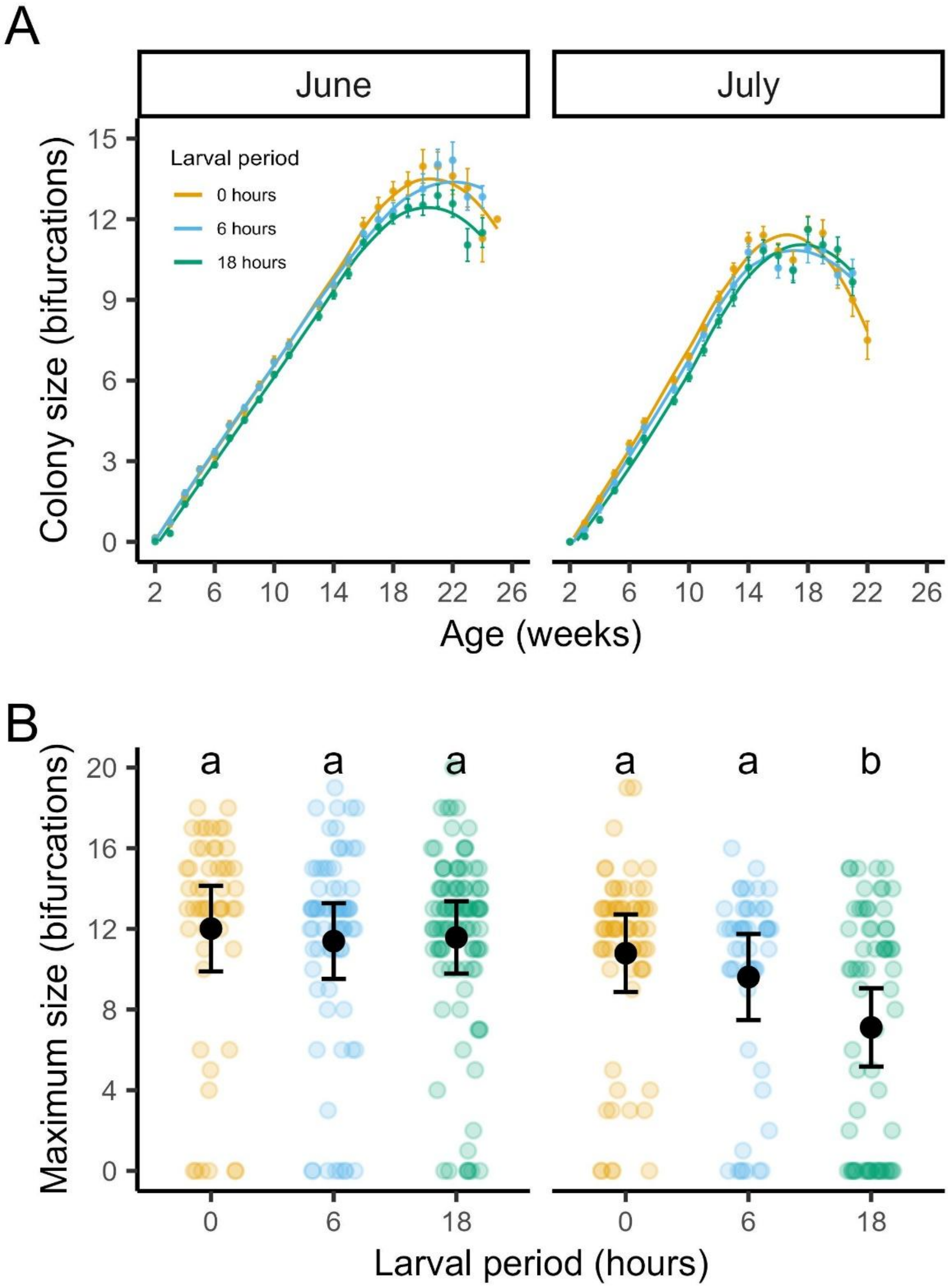

Figure S3. The effect of larval period on (A) weekly growth and (B) the maximum size of Bugula neritina. In panel A coloured dots show the mean estimate and vertical lines show 95% confidence interval. The coloured line is line of best fit. There was no data collected in week 12 for June and week 8 for July due to bad weather. In panel B coloured dots show the maximum size of individual colonies and black dots show the mean with 95% confidence intervals. Letters indicate where significant differences are observed between larval periods. Bifurcations (n) relates to zooid number (z) according to the equation: $z = 2^{n+2}$ (Keough & Chernoff, 1987).

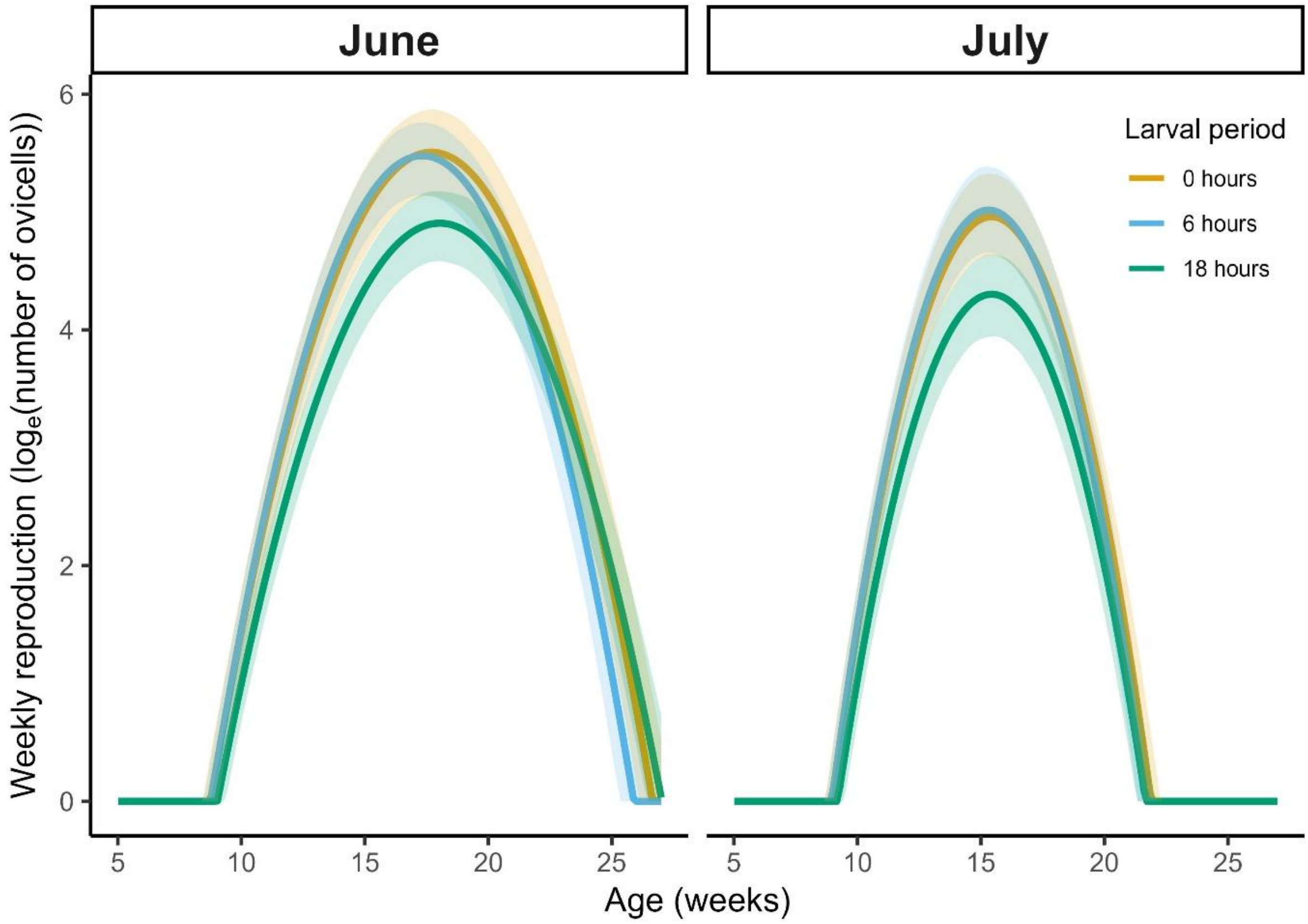

Figure S4. The effect of larval period on the estimated reproductive trajectory of *Bugula neritina* colonies on the log scale. Coloured lines show the estimated reproduction of individual colonies derived from quadratic models of reproductive output. The shaded area shows the 95% confidence interval for the estimate generated from 500 parametric bootstrap simulations.

Literature cited:

Keough, M. J., & Chernoff, H. (1987). Dispersal and Population Variation in the Bryozoan Bugula Neritina. Ecology, 68(1), 199-210. https://doi.org/https://doi.org/10.2307/1938820

Kosman, E. T., & Pernet, B. (2009). Diel Variation in the Sizes of Larvae of Bugula neritina in Field Populations. *The Biological Bulletin*, *216*(1), 85-93. https://doi.org/10.1086/BBLv216n1p85

Lemaître, J.-F., Moorad, J., Gaillard, J.-M., Maklakov, A. A., & Nussey, D. H. (2024). A unified framework for evolutionary genetic and physiological theories of aging. PLOS Biology, 22(2), e3002513. https://doi.org/10.1371/journal.pbio.3002513
